# Reduced Dynamic Models in Epithelial Transport

**DOI:** 10.1155/2013/654543

**Published:** 2013-02-28

**Authors:** Julio A. Hernández

**Affiliations:** Sección Biofísica, Facultad de Ciencias, Universidad de la República, Iguá esq. Mataojo, 11400 Montevideo, Uruguay

## Abstract

Most models developed to represent transport across epithelia assume that the cell interior constitutes a homogeneous compartment, characterized by a single concentration value of the transported species. This conception differs significantly from the current view, in which the cellular compartment is regarded as a highly crowded media of marked structural heterogeneity. Can the finding of relatively simple dynamic properties of transport processes in epithelia be compatible with this complex structural conception of the cell interior? The purpose of this work is to contribute with one simple theoretical approach to answer this question. For this, the techniques of model reduction are utilized to obtain a two-state reduced model from more complex linear models of transcellular transport with a larger number of intermediate states. In these complex models, each state corresponds to the solute concentration in an intermediate intracellular compartment. In addition, the numerical studies reveal that it is possible to approximate a general two-state model under conditions where strict reduction of the complex models cannot be performed. These results contribute with arguments to reconcile the current conception of the cell interior as a highly complex medium with the finding of relatively simple dynamic properties of transport across epithelial cells.

## 1. Introduction

The transport of water and solutes across epithelia is a relevant physiological property of higher organisms. To perform transport, epithelial cells develop a polarized distribution of membrane molecules, which localize at distinct apical and basolateral domains of the plasma membrane [1, 2]. The analysis and interpretation of quantitative data about solute and water transport across epithelia have constituted a major objective of cell physiologists. The majority of the models classically developed to represent solute transport across epithelia have considered that the interior of the epithelial cells constitutes a well-stirred, homogeneous compartment, characterized by a single value of concentration of the transported species [3–5]. This view implicitly assumes that the intracellular diffusion coefficient of the species remains constant and that diffusion occurs freely and rapidly, so that the intracellular solute concentrations attain a single equilibrium value at a faster time scale than the overall process. This conception differs markedly from the current view about the structural and functional characteristics of the cell interior. In this conception, the intracellular compartment is regarded as a highly crowded media of marked structural and functional heterogeneity [6–8]. The effects of macromolecular crowding and structural organization on the activity of macromolecules and smaller dissolved species represent major topics for the understanding of the cellular behavior [9]. Realistic approaches to describe diffusion in cellular media require computational simulations that employ, for instance, Brownian dynamics [10, 11], finite-element methods [12, 13], or The Virtual Cell framework [14, 15].

Can the finding of relatively simple dynamic properties of transport processes in epithelia be compatible with the complex structural conception of the cell interior? The general objective of this work is to contribute with the basic aspects of one formal theoretical approach to answer this question. In particular, this study employs mathematical modeling to uncover properties that could be employed to measure structural cellular complexity. Since a detailed computer simulation of the solute movement throughout the intracellular medium would, although more realistic, not be easy to incorporate in a representation of the overall transport process, this study adopts a simpler approach which, nevertheless, may provide some basic conclusions. In this way, as an alternative to explicit computational simulations of the intracellular media, this study assumes that the unidirectional solute movement can approximately be represented by a discrete, multicompartment model. To be noted, discrete approaches to describe flow through nonhomogeneous media have been employed to understand the basic aspects of percolation [16]. Similarly, the simple approach adopted here represents an initial attempt to reconcile macroscopic physiological evidence with microscopic cellular complexity. The multicompartment representation permits to express the transition of the solute between adjacent intracellular compartments via kinetic expressions; the overall dynamics are, therefore, governed by a system of linear differential equations. Multicompartmental strategies have been utilized, for instance, to understand the role of diffusion in brain processes [17] and to describe sarcomeric calcium movement [18].

In essence, the findings of this theoretical study suggest that the basic processes of transcellular transport across an epithelial cell between the two extracellular compartments may be reduced to an equivalent two-state linear model. The strategy of model reduction represents an alternative to study discrete systems with a high degree of complexity, such as biochemical networks, and permits to derive models that retain some of the relevant system properties under specific conditions. In this respect, linear systems of a relatively large number of components can be handled in a rather straightforward fashion. Thus, in macromolecular systems the reduction of linear intermediate transitions of multistate diagrams to yield simpler models has provided a tool, for instance, to understand the finding of simple kinetic behaviors in complex membrane transport systems [19]. In epithelial transport, nonlinearity may emerge as a consequence of interactions between different transported species or from the existence of feedback mechanisms, such as those involved in crosstalk responses [20–22]. The loss of linearity underlies the emergence of more complex behaviors of multistate systems and their reduction may inevitably require the design of alternative computational strategies [23]. In the present work, only the basic aspects of transcellular transport across epithelial cells are considered, which permit to conform a linear model with an arbitrary number of intermediate intracellular states. In this study, techniques analogous to the ones utilized for linear macromolecular kinetics are employed to obtain reduced two-state models from the original multistate ones [19]. The numerical simulations performed here also permit to obtain the noteworthy result that, under conditions where strict model reduction does not occur, an equivalent pseudo-two-state dynamic model can nevertheless be approximated. These results contribute with some arguments to reconcile the current conception of the cell interior as a highly complex media with the finding of relatively simple dynamic properties of transport across epithelial cells.

## 2. Models of Transcellular Transport of Solutes across Epithelial Cells

**Figure 1:**
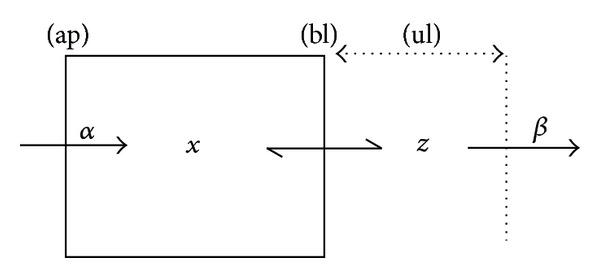
Scheme of an epithelial cell performing transcellular transport of a solute. The solute enters the cell at the apical membrane (ap) via active transport at rate *α* and exits at the basolateral membrane (bl) to an adjacent unstirred layer (ul), from which it is extracted at rate *β*. One of the simplest situations is represented, where the cell interior is assumed to be a homogeneous compartment characterized by a single value of solute concentration (*x*). The extracellular unstirred layer is also characterized by a single value of the solute concentration (*z*).

**Figure 2:**
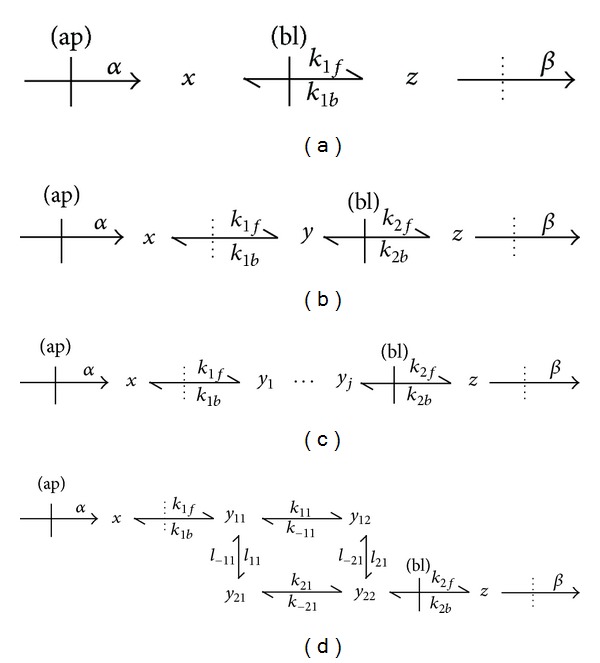
Different models of transcellular solute transport across an epithelial cell (cf. Figure 1). The dotted lines denote limits of unstirred layers (cf. Figure 1), the rest of the symbols as in Figure 1. The model in (a) (Model I) corresponds to the situation depicted in Figure 1. (b) shows a more complex model (Model II), where the solute distributes into two distinctive intracellular compartments (at concentrations *x* and *y*). In the general scheme of (c), the solute may distribute along a larger number of intracellular compartments (at concentrations *x* and *y*_1_ to *y*_*j*_). In particular, this work considers models having from two (Model III) to four (Model V) intermediate states “*y*.” (d) corresponds to a simple case of bidimensional intracellular distribution of the solute (Model VI).

One of the simplest models of transcellular transport of a solute across an epithelial cell (e.g., an intestinal cell) is depicted in Figure 1. In this scheme, a solute (e.g., glucose) is being driven inside the cell via an active transport system of the apical membrane (e.g., the Na-glucose cotransporter). Under physiological conditions, this transport system is assumed to operate irreversibly at rate *α*. In this model, the strict homogeneous condition of the cell applies; that is, the solute concentration *x* is the same throughout the whole intracellular compartment. The solute is driven out of the cell at the basal domain via a passive, reversible transport system (e.g., the glucose transporter (GLUT2)). In the model considered (Figure 1), the solute accumulates in the unstirred layer adjacent to the basal membrane at a concentration *z* and exits this compartment at rate *β*. Models exploring the possible role of unstirred layers at the extracellular cell surface in transcellular transport have been developed, for instance, to explain contradictory data about solute and solvent coupling in epithelia [24]. It is not the objective of this work to contribute to the discussion of the importance of unstirred layers in explaining quantitative data about epithelial solute and water transport, a matter that has received attention in the past [25], but to consider plausible models of transcellular transport of a single dissolved solute for illustrative purposes. An alternative to the meaning of the intermediate state *z* is to assume that it directly corresponds to the solute concentration at the apical extracellular compartment. In this case, *β* would represent its rate of extraction from other tissues. The elementary dynamic model governing the transport process described in Figure 1 (Model I) is shown in more detail in Figure 2(a) and is given by

(1) $$\begin{array}{c} x^{\prime }=\alpha -k_{1f}x+k_{1b}z,\\ z^{\prime }=k_{1f}x-\left(k_{1b}+\beta \right)z, \end{array}$$

where *x*′(*z*′) denotes the time derivative of *x*(*z*) and, *α*, *k*_1*f*_, *k*_1*b*_, and *β* are (positive) rate parameters. The solution and basic properties of this model are given in Appendix A, solely as a reference to the studies performed in this work. It can be easily concluded from the study of the explicit solution (Appendix A) or from the stability analysis (not shown) that the steady state of this model represents an asymptotically stable configuration, a characteristic property of integrated systems of membrane transport in general [26–29].

A somewhat more complex model can be obtained if one assumes, for instance, that an unstirred layer additionally exists at the intracellular surface of the apical membrane (Figure 2(b)). In this case, the solute accumulates at this layer at concentration *x* and then diffuses reversibly to the rest of the cell, where it achieves a uniform concentration *y*. As in the previous case, it is then transported reversibly to the extracellular space at the level of the basal domain. The corresponding dynamic model (Model II) is given by

(2) $$\begin{array}{c} x^{\prime }=\alpha -k_{1f}x\;+\;k_{1b}y,\\ y^{\prime }=k_{1f}x-\;\left(k_{1b}+k_{2f}\right)y\;+\;k_{2b}z,\\ z^{\prime }=k_{2f}y-\left(k_{2b}+\beta \right)z, \end{array}$$

where *α*, *k*_1*f*_, *k*_1*b*_, *k*_2*f*_, *k*_2*b*_, and *β* are rate parameters. Assuming that *y* is a quasistationary intermediate, the model given by (2) can be reduced to a simple two-state model formally analogous to Model I (Appendix B).

As mentioned above (Section 1), the present view about the intracellular compartment is far from the homogeneous, dilute perspective classically invoked to perform quantitative interpretations of cellular transport properties. A more realistic conception of the cell interior implies a highly crowded, heterogeneous media where instant equilibration to a unique intracellular concentration of a specific species may not represent a realistic approximation.  Figure 2(c) depicts a general model of unidirectional intracellular transport that assumes the existence of several intermediate internal compartments for the transported species. These successive compartments, extending to the rest of the cell starting from the unstirred layer at the intracellular apical domain, are characterized by specific concentrations (*y*_1_ to *y*_*j*_) of the transported species. The transitions between adjacent compartments are reversible and governed by first-order rate constants. In this work, we shall further consider models of the general type of Figure 2(c), ranging from two (i.e., *y*_1_, *y*_2_) to four (*y*_1_, …, *y*_4_) intermediate states, to perform some numerical studies (see below). These models shall be designated as Models III to V, respectively. An extension of the unidimensional model to more realistic situations would consider the inclusion of a larger number of intermediate states. Still further complexity is attainable if one assumes two-dimensional distribution of the intracellular solute. As an example, Figure 2(d) shows a situation where the solute is distributed inside the cell, apart from the unstirred layer at the apical membrane, into the simplest two-dimensional network of intermediate states (Model VI). More complex configurations in the two- and even three-dimensional domains are certainly conceivable, but their analysis would require the employment of alternative procedures, such as Monte Carlo simulations. Appendix B illustrates the procedures of linear model reduction [19, 30] employing Models II and VI as examples. It is shown there that, under some conditions, complex models of the type of Models II to VI can be reduced to a simple two-state model qualitatively similar to Model I ((A.1)). As examples, ((B.1)) and ((B.2)) give the expressions obtained for the reduced rate constants *r*_12_ and *r*_21_ ((A.1)) for the cases of Models II and VI, respectively.

In order to illustrate the concepts introduced here, the next section contains numerical studies of some dynamic properties of the models. Of particular interest is the finding that, under conditions not permitting strict reduction, the models nevertheless exhibit a dynamic behavior approximately equivalent to a two-state dynamic model governed by the general equations ((A.1)).

## 3. Numerical Results and Discussion

In this section, numerical studies are performed to compare the dynamic behaviors between the original and the reduced models, for the different models considered and for different values of some of the parameters. In essence, the procedure followed here consists in simulating the time courses of the model dynamics in response to perturbations from the steady state. The results shown are not exhaustive and only intended to illustrate some basic properties of the models. For this reason, the numerical values employed here for the rate constants are arbitrary and only results of the relative variations of the variable *z* with respect to the steady-state value (z*) are shown. For the choice of the numerical values, the only restrictive condition assumed was that the parameter *α* should have a larger value than the rest of the parameters, since it represents the rate of active transport of the solute (cf. Figure 1).

**Figure 3:**
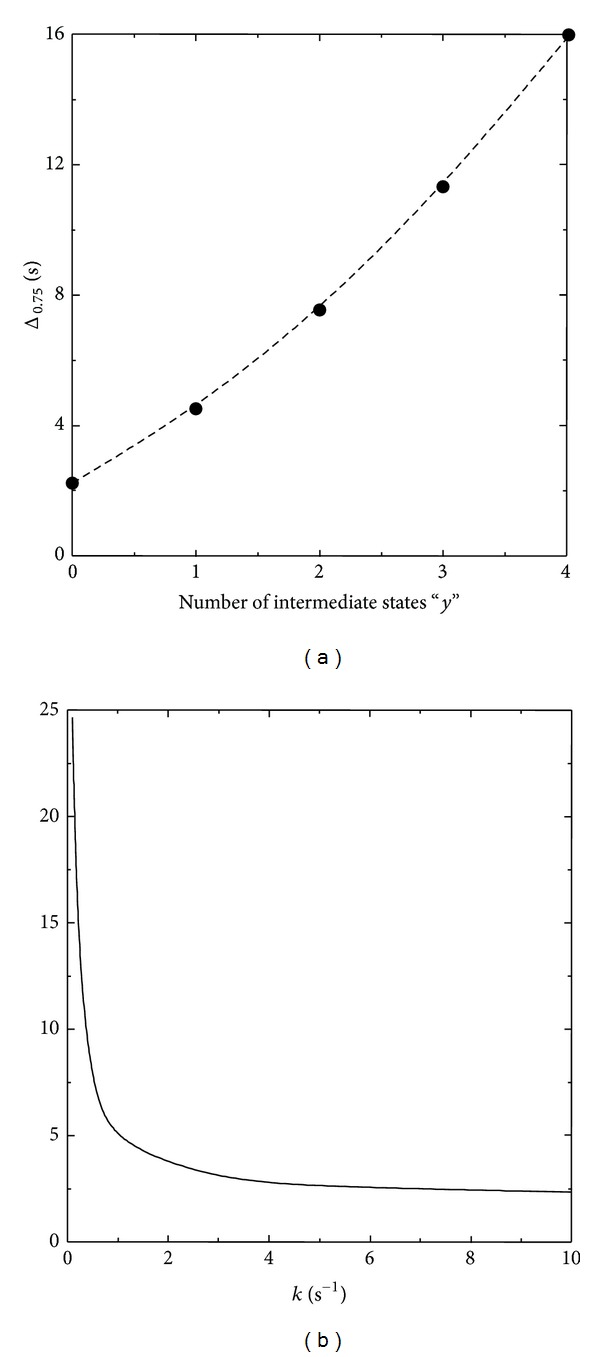
Plots of the time delay Δ_0.75_ versus (a) the number of intermediate states “*y*” in Models I to V for *k* = 1 and versus (b) *k* for the case of Model II (Δ_0.75_: time elapsed between *t* = 0 and *t* for *z*/z* = 0.75; *k* = *k*_1*f*_ = *k*_2*b*_, cf. Figure 2). The numerical integrations were performed employing the Runge-Kutta fourth-order method. For every run, for *t* = 0, *z*/z* = 0.5. For every case, *α* = 10 and the numerical value for the rest of the parameters was 1.

The increasing complexity of the models (i.e., from Model I towards Model V, Figure 2) in turn determines modifications in properties that may have physiological significance, such as the time delay to achieve the steady state from an initial perturbed condition.  Figure 3 shows the effect of increasing complexity and of the rate constants on the time delay to achieve the steady state. As expected, the increasing complexity (measured by the number of intermediate states “*y*,” Figure 3(a)) determines an increase in the time delay, while the rise in some of the intermediate rate constants produces the opposite effect (Figure 3(b)). Since the dynamic behavior of the complex model may be indistinguishable from that of a two-state model (Figures 1 and 2(a)), either by satisfying the conditions of model reduction or by approximate behavior (see below), measurements of the actual values of the time delays, if possible, may provide clues to infer the degree of structural complexity of the cellular transport system.

**Figure 4:**
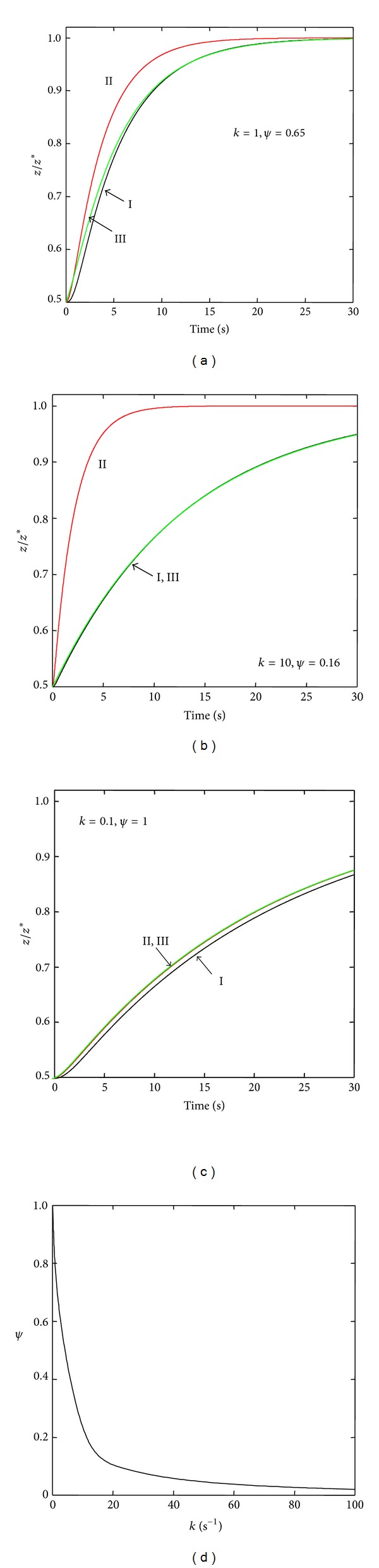
(a)–(c) Plots of *z*/z* versus time for the case of Model II and for different values of *k*. Numerical integrations: Curve I, (2); Curve II, ((A.1)) and ((B.1)). Direct numerical solutions: curve superposed to Curve I, ((A.2)); Curve III, ((A.6)). (d) Plot of *ψ* versus *k*. Numerical methods: numerical values of the rest of the parameters and definition of *k* are the same as in Figure 3. For each value of *k*, the numerical value for the adjust factor *ψ* (Appendix A) was obtained by trial and error in order to attain the best approximation to Curve I. The curve *ψ* versus *k* (d) was obtained as the best fit to a sample of values of *ψ* for the corresponding values of *k*, throughout the whole range of values of *k* considered.

**Figure 5:**
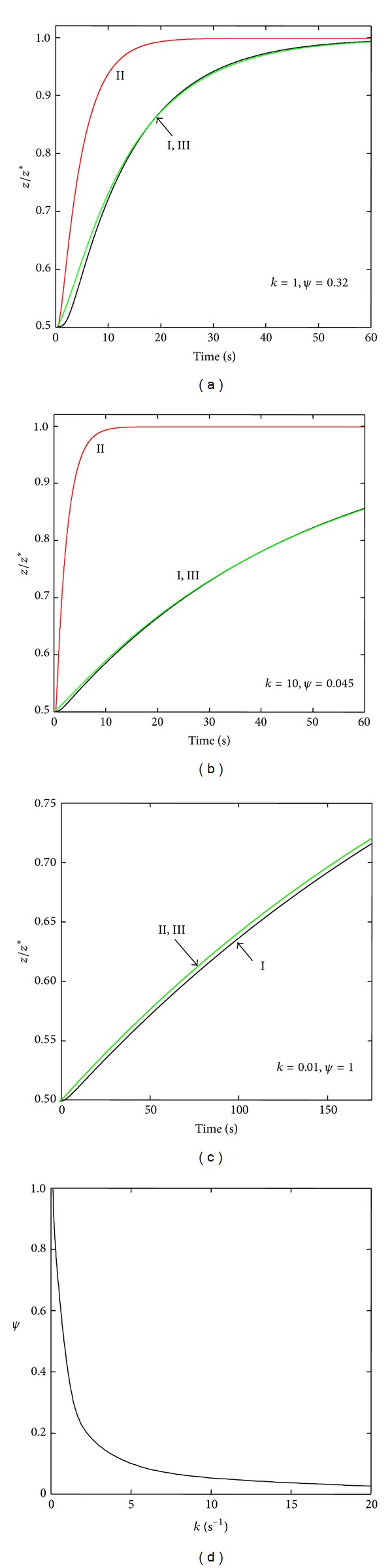
Similar to Figure 4, but for Model VI. In this case, Curve I corresponds to the numerical integration of the complete model shown in Figure 2(d) (explicit equations not shown in the text). In addition, ((A.1)) and ((B.2)) were employed to obtain Curve II.

Figures 4 and 5 show the dynamic responses of Models II and VI (Figure 2), respectively, to perturbations of the steady state. The figures display the numerical integrations of the complete models, the corresponding reduced models (((A.1)) and ((B.1)) for Model II and ((A.1)) and ((B.2)) for Model VI), and the approximations to the complete models ((A.6)). For the two models, the numerical integration of ((A.1)) yielded similar results to the direct numerical solution of ((A.2)). As can be seen in Figures 4 and 5, for parameter values satisfying the necessary reduction conditions (Appendix B), the strictly reduced models yield dynamic behaviors undistinguishable from the original ones (Figures 4(c) and 5(c)). The necessary conditions for model reduction may be somewhat unrealistic, however, since they imply the quasistationary hypothesis for the intermediate states (Appendix B). It is, therefore, a noteworthy result that, for values not satisfying the reduction conditions (Figures 4(a), 4(b), 5(a), and 5(b)), the numerical studies nevertheless permitted to approximate two-state models by the simple procedure of introducing an adjust factor Ψ to the time constants of the corresponding reduced models. This property, not further analyzed here, is possibly a consequence of the linear character of the model. As revealed by Figures 4(d) and 5(d), for large values of *k* (i.e., far from the reduction conditions) low values of Ψ are required to obtain a proper approximation to the original model behavior. The figures also show that, in order to obtain that approximation, Ψ tends to unity as *k* tends to zero. Thus, Ψ may represent a measurement of the degree of complexity of the original model, since its value depends on how distant the actual model dynamics are from the reduction conditions.

Similar results to the ones displayed in Figures 4 and 5 were obtained for Models III to V (not shown). In particular, in every case it was possible to empirically approximate a two-state model to the complete one when strict conditions for model reduction did not apply. Simulations performed for different values of the intermediate rate parameters (although conserving the rule that *α* should be larger than the rest of the parameters), also permitted to obtain reasonable approximations to Model II employing ((A.6)) (not shown). These results suggest that, at least for the case of some processes of epithelial transport of solutes, it is possible to describe these processes by a relatively simple model of the general type given by ((A.1)). However, the results of this work also suggest that, under these circumstances, it is not possible to conclude that the actual underlying process strictly corresponds to the simple model described in Figure 1, characterized by a unique intracellular concentration of the solute. The actual intracellular distribution of the solute may be approximated by a more complex configuration, such as the ones represented by Models II to VI (Figure 2), or still more complex. As mentioned in Section 1, it must be emphasized that a discrete multicompartment description of the intracellular compartment can only be considered as an initial approach to represent this highly complex medium, an approach that may nevertheless be operative for the consideration of some specific issues, such as the one addressed in this study.

## 4. Conclusions

The results of this theoretical study permit to suggest that complex models of transepithelial transport of solutes may nevertheless exhibit dynamic properties undistinguishable from those of simple models. At least in the realm of linear models of transport, it was shown here that models incorporating several intermediate states of the solute in the intracellular compartment may, under the proper conditions, be reduced to simple two-state models that assume the existence of a unique concentration value for the intracellular solute. Even if those reduction conditions are not accomplished, the numerical studies also permitted to obtain approximate two-state dynamic models to mimic the original complex ones. Taken together, the results of this work permit to ascertain that, at least for the case of the elementary processes of epithelial transport of solutes, it may be possible to reconcile the finding of relatively simple transcellular transport dynamics with the current conception of the cell interior as a highly complex structural media.

This work has, therefore, focused on the case of the transport of solutes across epithelial cells to illustrate that complex models of transport can exhibit dynamic behaviors undistinguishable from those of simple ones. The occasional emergence of relatively simple dynamic properties may be a property encountered for the case of many other complex biological processes, such as transitions between macromolecular states [19] and reactions in biochemical networks [23]. As illustrated in this work, at least in some cases, a possible means to understand the emergence of relatively simple behaviors in complex dynamical systems can be obtained in a rather straightforward fashion by employing standard techniques of reduction of dynamic models.

## Appendices

### A. Solution of the General Two-State Model of Transepithelial Solute Transport

As a reference to this study, this section resumes the basic properties of the general two-state model of transepithelial solute transport. This model is given by

(A.1) $$\begin{array}{c} \frac{dx}{dt}=\alpha \;-\;r_{12}x\;+\;r_{21}z,\\ \frac{dz}{dt}=r_{12}x\;-\;\left(r_{21}+\beta \right)z, \end{array}$$

where *α* and *β* have the same meanings for all the models (Figures 1 and 2) and where the *r*'s are, in general, reduced rate constants (see Appendix B). For the particular case of the simplest model ((1), Model I), *r*_12_ = *k*_1*f*_ and *r*_21_ = *k*_1*b*_.

The solution of the system given by (A.1) can be obtained employing standard procedures. For the case of *z*, the solution reads

(A.2) $$\begin{array}{c} z\left(t\right)=z^{*}+C_{1}\exp\left(m_{1}t\right)+C_{2}\exp\left(m_{2}t\right), \end{array}$$

where *z** is the steady-state value of *z*[*z** = *α*/*β*] and where

(A.3) m1,2=[  −  Φ±(Δ)1/2]2,with  Φ=(r12+r21+β),Δ=Φ2−4r12β.

Since Φ > (Δ)^1/2^,  *m*_1_ and *m*_2  _are necessarily negative. Hence, from any initial value of *z*, the system given by A.1 converges asymptotically to the steady-state value *z**.

For the case that *z*(0) = *z**/2,

(A.4) $$\begin{array}{c} C_{1}=-\;\left(\frac{z^{*}}{2}\right)\left(1+K\right),\;\;C_{2}=\left(\frac{z^{*}}{2}\right)\;K,\\ \text{With}\;K=\frac{\left[m_{1}\left(1+m_{1}\right)+r_{12}\beta \right]}{\left\{\Phi \left[m_{2}\left(1+m_{2}\right)\;-\;m_{1}\left(1\;+\;m_{1}\right)\right]\right\}}. \end{array}$$

In the numerical studies, an approximate solution to the dynamics of a complete (i.e., nonreduced) model can be obtained by introducing an adjust factor Ψ to the parameters *m*_1_ and *m*_2_ of the corresponding reduced model:

(A.5) $$\begin{array}{c} \mu_{1}=\Psi m_{1},\;\;\mu_{2}=\Psi m_{2}\;. \end{array}$$

The approximate solution reads

(A.6) $$\begin{array}{c} z\left(t\right)=z^{*}+C_{1}\exp\left(\mu_{1}t\right)+C_{2}\exp\left(\mu_{2}t\right), \end{array}$$

where *C*_1_ and *C*_2_ are the same as in the corresponding reduced model (A.2).

### B. Reduction of Linear Dynamic Models of Transepithelial Transport

This section summarizes the procedure to reduce dynamic models of transcellular transport, for the case that some states of the transported species are transient intermediates. The method described in this section is based upon the techniques originally developed by Hill [30] for the reduction of linear sequences of transitions in biochemical systems and further extended to more complex configurations [19]. Instead of deriving general expressions, the procedure is illustrated employing Models II and VI as examples (Figures 2(b) and 2(d)). The method described here can be adapted in a straightforward manner to handle more complicated schemes. For a more detailed exposition of the reduction technique, the reader may consult [19].

For the case of Model II, if *y* is a transient intermediate ((2)), we may assume that, at any time, *dy*/*dt* = 0. This requires that *k*_1*b*_ ≫ *k*_1*f*_ and *k*_2*f*_ ≫ *k*_2*b*_. From this condition, (2) can be transformed into a system of the form of (A.1), with the reduced constants *r*_12_ and *r*_21_ given in this case by

(B.1) $$\begin{array}{c} r_{12}\;=\frac{k_{1f}\;k_{2f}}{\left(k_{1b}+k_{2f}\right)},\;\;r_{21}\;=\;\frac{k_{1b}\;k_{2b}}{\left(k_{1b}+k_{2f}\right)}. \end{array}$$

Analogously, the more complex models (Models III to VI) can be reduced to the general two-state model given by (A.1) under the condition that all the states “*y*” are transient intermediates. For example, for the case of Model VI (Figure 2(d)) this is achieved if the rate constants *k*_1*f*_ and *k*_2*b*_ are significantly smaller than the other constants. The expressions for the reduced rate constants *r*_12_ and *r*_21_ that can be obtained for Model VI under this condition are the following (cf. Figure 2(d)):

(B.2) $$\begin{array}{c} r_{12}\;=\frac{D_{12}}{D},\;\;r_{21}\;=\frac{D_{21}}{\;D} \end{array}$$

with

(B.3) D12  =  k1fk2f[k11l21(k21+l−11)+  l11k21(l21+k−11  )],D21  =k1b  k2b[k−11l−21(k21+l−11)+  l−11k−21(l21+k−11)],D  =(k1b  k2f)(k21+  l−11)(l21+k−11)+  k1b[k−11l−11(k−21+l−21)+l−11l21k−21+  k−11k21l−21]+k2f[k21  l21(k11+l11)+  k11l−11l21+  k−11k21l11].
